# 2-{4-[(Quinolin-8-yl­oxy)meth­yl]phen­yl}benzonitrile

**DOI:** 10.1107/S1600536811012724

**Published:** 2011-04-29

**Authors:** Bin Wei

**Affiliations:** a Ordered Matter Science Research Center, Southeast University, Nanjing 210096, People’s Republic of China

## Abstract

In the title compound, C_23_H_16_N_2_O, the bond angle at the O atom that connects the benzene ring and the quinoline ring system is 116.0 (2)°. The quinoline ring system make a dihedral angle of 16.5 (2)° with the adjacent benzene ring. The dihedral angle between the biphenyl benzene rings is 70.8 (2)°.

## Related literature

For background to tetra­zoles, see: Hang *et al.* (2009 ▶). For our investigation of tetra­zole compounds and their coordination modes, see: Xiong *et al.* (2002 ▶). For the preparation of tetra­zoles using *in situ* synthesis of tetra­zole through cyclo­addition between organotin azide and organic cyano groups, see: Chen *et al.* (2010 ▶); Ye *et al.* (2006 ▶).
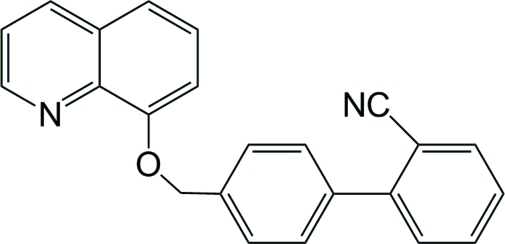


## Experimental

### 

#### Crystal data

C_23_H_16_N_2_O
*M* *_r_* = 336.38
Orthorhombic, 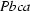

*a* = 14.526 (4) Å
*b* = 8.957 (3) Å
*c* = 27.126 (8) Å
*V* = 3529.3 (18) Å^3^
*Z* = 8
Mo *K*α radiation
μ = 0.08 mm^−1^
*T* = 293 K
0.20 × 0.20 × 0.20 mm

#### Data collection

Rigaku Mercury CCD diffractometer
Absorption correction: multi-scan (*CrystalClear*; Rigaku, 2005) *T*_min_ = 0.842, *T*_max_ = 1.000
36455 measured reflections
4036 independent reflections
2865 reflections with *I* > 2σ(*I*)
*R*_int_ = 0.073

#### Refinement

*R*[*F* ^2^ > 2σ(*F* ^2^)] = 0.082
*wR*(*F* ^2^) = 0.222
*S* = 1.24
4036 reflections
235 parameters
H-atom parameters constrained
Δρ_max_ = 0.19 e Å^−3^
Δρ_min_ = −0.22 e Å^−3^

### 

Data collection: *CrystalClear* (Rigaku, 2005 ▶); cell refinement: *CrystalClear*; data reduction: *CrystalClear*; program(s) used to solve structure: *SHELXS97* (Sheldrick, 2008 ▶); program(s) used to refine structure: *SHELXL97* (Sheldrick, 2008 ▶); molecular graphics: *SHELXTL* (Sheldrick, 2008 ▶); software used to prepare material for publication: *SHELXL97*.

## Supplementary Material

Crystal structure: contains datablocks I, global. DOI: 10.1107/S1600536811012724/jh2278sup1.cif

Structure factors: contains datablocks I. DOI: 10.1107/S1600536811012724/jh2278Isup2.hkl

Additional supplementary materials:  crystallographic information; 3D view; checkCIF report

## Figures and Tables

**Fig. 1.**
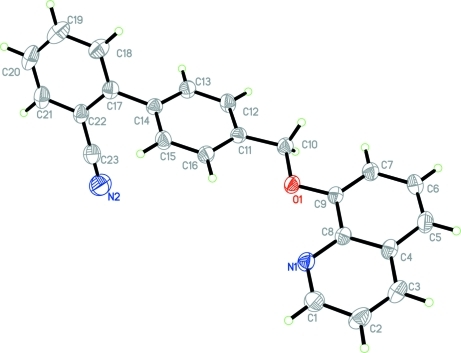
Crystal structure of the title compound with labelling and displacement ellipsoids drawn at the 30% probability level.

**Fig. 2.**
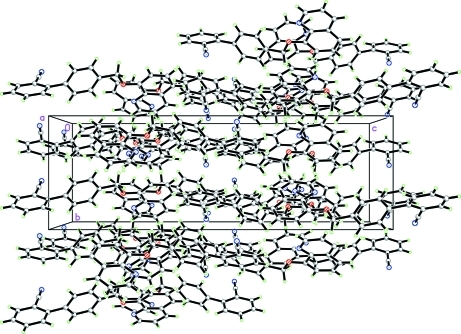
Crystal structure of the title compound with view along the a axis. Intermolecular interactions are shown as dashed lines.

## supplementary crystallographic information

### Comment

Tetrazole compounds have been studied for more than one hundred years and applied in various areas (Hang *et al.*, 2009). As a part of systematic investigation of new tetrazole compounds and discovery of new coordination mode (Xiong *et al.*, 2002), we get the synthesis of the title compound C22 H16 N2 O,and preparation of tetrazoles *in situ* synthesis of tetrazole through cycloaddition between organotin azide and organic cyano group (Ye *et al.*, 2006; Chen *et al.*, 2010).

In the asymmetric unit of the title compound, the planes angle between the two benzene rings is 70.8°. O1 connect quinoline ring and sartan ring with a 115.9 bond-angle and the bond length O1—C10 is 1.4261 (35) Å, O1—C9 is 1.3691 (33) Å). The quinoline ring make a small dihedral angle of 16.5° with adjacent benzene ring (Fig 1). Fig 2 shows that the molecules assemble as straight chain in the crystal structure along the *a* axis.

### Experimental

8-hydroxyquinoline(1.45 g,10 mmol) was added in a solution of 4'-Bromoethyl-2-cyanobiphenyl(2.71 g,10 mmol) in methanol(20 ml).After the mixture was stirred for 10 h at 355 K,the precipitate was filtered off and the solution was evaporated in vacuum. The crude product was then crystallized form ethanol to yield colourless prisms of the title compound.

### Refinement

H atoms were positioned geometrically, with C—H = 0.93 and 0.97 Å for aromatic and methylene H respectively, and constrained to ride on their parent atoms with Uĩso~(H) = xU~eq~(C), where *x* = 1.5 for methyl H and *x* = 1.2 for all other H atoms.

### Crystal data

**Table:**

C_23_H_16_N_2_O	*D*_x_ = 1.266 Mg m^−^^3^
*M**_r_* = 336.38	Mo *K*α radiation, λ = 0.71073 Å
Orthorhombic, *P**b**c**a*	Cell parameters from 6593 reflections
*a* = 14.526 (4) Å	θ = 2.3–27.5°
*b* = 8.957 (3) Å	µ = 0.08 mm^−^^1^
*c* = 27.126 (8) Å	*T* = 293 K
*V* = 3529.3 (18) Å^3^	Prism, colorless
*Z* = 8	0.20 × 0.20 × 0.20 mm
*F*(000) = 1408	

### Data collection

**Table:**

Rigaku Mercury CCD diffractometer	4036 independent reflections
Radiation source: fine-focus sealed tube	2865 reflections with *I* > 2σ(*I*)
graphite	*R*_int_ = 0.073
Detector resolution: 28.5714 pixels mm^-1^	θ_max_ = 27.5°, θ_min_ = 1.5°
ω scans	*h* = −18→18
Absorption correction: multi-scan (*CrystalClear*; Rigaku, 2005)	*k* = −11→11
*T*_min_ = 0.842, *T*_max_ = 1.000	*l* = −35→35
36455 measured reflections	

### Refinement

**Table:**

Refinement on *F*^2^	Secondary atom site location: difference Fourier map
Least-squares matrix: full	Hydrogen site location: inferred from neighbouring sites
*R*[*F*^2^ > 2σ(*F*^2^)] = 0.082	H-atom parameters constrained
*wR*(*F*^2^) = 0.222	*w* = 1/[σ^2^(*F*_o_^2^) + (0.0817*P*)^2^ + 0.8691*P*] where *P* = (*F*_o_^2^ + 2*F*_c_^2^)/3
*S* = 1.24	(Δ/σ)_max_ < 0.001
4036 reflections	Δρ_max_ = 0.19 e Å^−^^3^
235 parameters	Δρ_min_ = −0.22 e Å^−^^3^
0 restraints	Extinction correction: *SHELXL*
Primary atom site location: structure-invariant direct methods	Extinction coefficient: 0.0000

### Special details

**Table:**

Geometry. All esds (except the esd in the dihedral angle between two l.s. planes) are estimated using the full covariance matrix. The cell esds are taken into account individually in the estimation of esds in distances, angles and torsion angles; correlations between esds in cell parameters are only used when they are defined by crystal symmetry. An approximate (isotropic) treatment of cell esds is used for estimating esds involving l.s. planes.
Refinement. Refinement of F^2^ against ALL reflections. The weighted R-factor wR and goodness of fit S are based on F^2^, conventional R-factors R are based on F, with F set to zero for negative F^2^. The threshold expression of F^2^ > 2sigma(F^2^) is used only for calculating R-factors(gt) etc. and is not relevant to the choice of reflections for refinement. R-factors based on F^2^ are statistically about twice as large as those based on F, and R- factors based on ALL data will be even larger.

### Fractional atomic coordinates and isotropic or equivalent isotropic displacement parameters (Å^2^)

**Table:**

	*x*	*y*	*z*	*U*_iso_*/*U*_eq_	
O1	0.28089 (13)	0.1887 (2)	0.29588 (6)	0.0503 (5)	
N1	0.43511 (16)	0.3299 (3)	0.26974 (8)	0.0494 (6)	
C8	0.38445 (18)	0.2633 (3)	0.23340 (9)	0.0421 (6)	
C11	0.17557 (18)	0.1423 (3)	0.36270 (10)	0.0430 (6)	
C10	0.2001 (2)	0.1096 (3)	0.30999 (10)	0.0501 (7)	
H10A	0.2103	0.0032	0.3060	0.060*	
H10B	0.1494	0.1383	0.2887	0.060*	
C9	0.30312 (19)	0.1836 (3)	0.24685 (9)	0.0435 (6)	
C4	0.4091 (2)	0.2693 (3)	0.18282 (10)	0.0481 (7)	
C16	0.2179 (2)	0.2507 (4)	0.39095 (10)	0.0534 (7)	
H16A	0.2675	0.3036	0.3781	0.064*	
C17	0.0766 (2)	0.2387 (3)	0.50852 (10)	0.0502 (7)	
C14	0.11330 (18)	0.2051 (3)	0.45827 (10)	0.0462 (6)	
C7	0.2541 (2)	0.1086 (3)	0.21157 (11)	0.0542 (7)	
H7A	0.2025	0.0537	0.2207	0.065*	
C5	0.3561 (2)	0.1925 (4)	0.14748 (11)	0.0608 (8)	
H5A	0.3727	0.1960	0.1144	0.073*	
C22	0.1263 (2)	0.2008 (3)	0.55117 (10)	0.0540 (7)	
C15	0.1872 (2)	0.2815 (4)	0.43839 (10)	0.0566 (8)	
H15A	0.2167	0.3544	0.4570	0.068*	
C13	0.0722 (2)	0.0939 (3)	0.42995 (11)	0.0536 (7)	
H13A	0.0231	0.0399	0.4428	0.064*	
C12	0.1033 (2)	0.0629 (3)	0.38308 (10)	0.0534 (7)	
H12A	0.0753	−0.0125	0.3649	0.064*	
C6	0.2808 (2)	0.1134 (4)	0.16163 (11)	0.0627 (9)	
H6A	0.2465	0.0620	0.1382	0.075*	
C2	0.5370 (2)	0.4211 (4)	0.20652 (13)	0.0682 (9)	
H2A	0.5888	0.4776	0.1988	0.082*	
C3	0.4879 (2)	0.3538 (4)	0.17066 (11)	0.0592 (8)	
H3A	0.5059	0.3630	0.1379	0.071*	
C1	0.5086 (2)	0.4045 (4)	0.25572 (12)	0.0626 (8)	
H1A	0.5442	0.4492	0.2801	0.075*	
C23	0.2152 (3)	0.1308 (4)	0.54694 (12)	0.0694 (9)	
C21	0.0894 (3)	0.2274 (4)	0.59785 (12)	0.0677 (9)	
H21A	0.1226	0.2013	0.6259	0.081*	
C18	−0.0090 (2)	0.3026 (4)	0.51433 (13)	0.0685 (9)	
H18A	−0.0434	0.3274	0.4866	0.082*	
C20	0.0046 (3)	0.2917 (4)	0.60239 (14)	0.0755 (11)	
H20A	−0.0200	0.3093	0.6335	0.091*	
N2	0.2850 (3)	0.0735 (5)	0.54410 (13)	0.0980 (12)	
C19	−0.0448 (3)	0.3306 (4)	0.56077 (16)	0.0808 (11)	
H19A	−0.1021	0.3756	0.5639	0.097*	

### Atomic displacement parameters (Å^2^)

**Table:**

	*U*^11^	*U*^22^	*U*^33^	*U*^12^	*U*^13^	*U*^23^
O1	0.0526 (11)	0.0582 (12)	0.0403 (10)	−0.0130 (9)	0.0084 (8)	−0.0045 (8)
N1	0.0530 (14)	0.0500 (13)	0.0452 (13)	−0.0064 (11)	0.0065 (11)	−0.0081 (10)
C8	0.0484 (15)	0.0366 (13)	0.0413 (14)	0.0055 (11)	0.0039 (12)	−0.0010 (10)
C11	0.0430 (14)	0.0442 (14)	0.0419 (13)	−0.0008 (12)	0.0014 (11)	0.0029 (11)
C10	0.0508 (16)	0.0548 (17)	0.0446 (15)	−0.0085 (13)	0.0050 (12)	−0.0010 (12)
C9	0.0477 (15)	0.0438 (14)	0.0390 (13)	0.0023 (12)	0.0022 (11)	−0.0024 (11)
C4	0.0576 (17)	0.0451 (15)	0.0417 (14)	0.0078 (13)	0.0067 (13)	−0.0003 (11)
C16	0.0488 (16)	0.0679 (18)	0.0434 (15)	−0.0165 (14)	0.0076 (12)	0.0006 (13)
C17	0.0560 (17)	0.0472 (15)	0.0475 (16)	−0.0096 (13)	0.0073 (13)	0.0010 (12)
C14	0.0454 (15)	0.0493 (15)	0.0439 (14)	0.0000 (12)	0.0026 (12)	0.0031 (12)
C7	0.0549 (16)	0.0600 (18)	0.0477 (15)	−0.0070 (14)	0.0022 (14)	−0.0087 (13)
C5	0.071 (2)	0.073 (2)	0.0380 (15)	0.0056 (17)	0.0026 (14)	−0.0006 (14)
C22	0.0641 (19)	0.0542 (16)	0.0438 (15)	−0.0110 (15)	0.0077 (14)	−0.0029 (13)
C15	0.0585 (18)	0.0662 (19)	0.0452 (16)	−0.0195 (15)	0.0037 (13)	−0.0071 (14)
C13	0.0552 (17)	0.0577 (17)	0.0479 (15)	−0.0166 (14)	0.0076 (13)	0.0020 (13)
C12	0.0611 (18)	0.0536 (16)	0.0455 (15)	−0.0181 (14)	0.0011 (14)	−0.0025 (13)
C6	0.066 (2)	0.077 (2)	0.0458 (16)	−0.0022 (17)	−0.0065 (15)	−0.0132 (15)
C2	0.070 (2)	0.0615 (19)	0.073 (2)	−0.0180 (17)	0.0265 (18)	−0.0058 (17)
C3	0.072 (2)	0.0546 (17)	0.0515 (17)	−0.0007 (16)	0.0188 (16)	0.0033 (14)
C1	0.0637 (19)	0.0588 (18)	0.065 (2)	−0.0146 (16)	0.0060 (16)	−0.0129 (15)
C23	0.075 (2)	0.089 (3)	0.0438 (17)	0.001 (2)	−0.0009 (17)	0.0068 (16)
C21	0.089 (3)	0.066 (2)	0.0479 (17)	−0.0169 (19)	0.0109 (17)	−0.0081 (15)
C18	0.065 (2)	0.074 (2)	0.066 (2)	0.0056 (18)	0.0153 (17)	0.0038 (17)
C20	0.103 (3)	0.060 (2)	0.063 (2)	−0.014 (2)	0.036 (2)	−0.0132 (17)
N2	0.088 (3)	0.135 (3)	0.071 (2)	0.025 (2)	−0.0001 (19)	0.015 (2)
C19	0.080 (3)	0.070 (2)	0.092 (3)	0.005 (2)	0.036 (2)	−0.002 (2)

### Geometric parameters (Å, °)

**Table:**

O1—C9	1.369 (3)	C5—C6	1.359 (5)
O1—C10	1.423 (3)	C5—H5A	0.9300
N1—C1	1.316 (4)	C22—C21	1.395 (4)
N1—C8	1.367 (3)	C22—C23	1.441 (5)
C8—C4	1.419 (4)	C15—H15A	0.9300
C8—C9	1.427 (4)	C13—C12	1.378 (4)
C11—C16	1.381 (4)	C13—H13A	0.9300
C11—C12	1.383 (4)	C12—H12A	0.9300
C11—C10	1.503 (4)	C6—H6A	0.9300
C10—H10A	0.9700	C2—C3	1.349 (5)
C10—H10B	0.9700	C2—C1	1.405 (4)
C9—C7	1.369 (4)	C2—H2A	0.9300
C4—C5	1.409 (4)	C3—H3A	0.9300
C4—C3	1.410 (4)	C1—H1A	0.9300
C16—C15	1.390 (4)	C23—N2	1.139 (5)
C16—H16A	0.9300	C21—C20	1.365 (5)
C17—C18	1.378 (4)	C21—H21A	0.9300
C17—C22	1.405 (4)	C18—C19	1.385 (5)
C17—C14	1.494 (4)	C18—H18A	0.9300
C14—C15	1.383 (4)	C20—C19	1.382 (6)
C14—C13	1.393 (4)	C20—H20A	0.9300
C7—C6	1.410 (4)	C19—H19A	0.9300
C7—H7A	0.9300		
			
C9—O1—C10	116.0 (2)	C21—C22—C23	119.4 (3)
C1—N1—C8	116.8 (2)	C17—C22—C23	120.0 (3)
N1—C8—C4	123.0 (2)	C14—C15—C16	120.8 (3)
N1—C8—C9	118.6 (2)	C14—C15—H15A	119.6
C4—C8—C9	118.4 (2)	C16—C15—H15A	119.6
C16—C11—C12	118.5 (3)	C12—C13—C14	120.8 (3)
C16—C11—C10	124.0 (2)	C12—C13—H13A	119.6
C12—C11—C10	117.4 (2)	C14—C13—H13A	119.6
O1—C10—C11	110.8 (2)	C13—C12—C11	120.9 (3)
O1—C10—H10A	109.5	C13—C12—H12A	119.5
C11—C10—H10A	109.5	C11—C12—H12A	119.5
O1—C10—H10B	109.5	C5—C6—C7	120.6 (3)
C11—C10—H10B	109.5	C5—C6—H6A	119.7
H10A—C10—H10B	108.1	C7—C6—H6A	119.7
O1—C9—C7	124.9 (3)	C3—C2—C1	118.8 (3)
O1—C9—C8	115.3 (2)	C3—C2—H2A	120.6
C7—C9—C8	119.8 (2)	C1—C2—H2A	120.6
C5—C4—C3	123.1 (3)	C2—C3—C4	120.0 (3)
C5—C4—C8	120.1 (3)	C2—C3—H3A	120.0
C3—C4—C8	116.8 (3)	C4—C3—H3A	120.0
C11—C16—C15	120.7 (3)	N1—C1—C2	124.5 (3)
C11—C16—H16A	119.6	N1—C1—H1A	117.7
C15—C16—H16A	119.6	C2—C1—H1A	117.7
C18—C17—C22	118.0 (3)	N2—C23—C22	178.8 (4)
C18—C17—C14	120.7 (3)	C20—C21—C22	120.0 (3)
C22—C17—C14	121.3 (3)	C20—C21—H21A	120.0
C15—C14—C13	118.2 (3)	C22—C21—H21A	120.0
C15—C14—C17	122.2 (3)	C17—C18—C19	121.2 (4)
C13—C14—C17	119.6 (2)	C17—C18—H18A	119.4
C9—C7—C6	120.9 (3)	C19—C18—H18A	119.4
C9—C7—H7A	119.5	C21—C20—C19	120.0 (3)
C6—C7—H7A	119.5	C21—C20—H20A	120.0
C6—C5—C4	120.2 (3)	C19—C20—H20A	120.0
C6—C5—H5A	119.9	C20—C19—C18	120.2 (4)
C4—C5—H5A	119.9	C20—C19—H19A	119.9
C21—C22—C17	120.6 (3)	C18—C19—H19A	119.9
			
C1—N1—C8—C4	1.3 (4)	C18—C17—C22—C23	178.7 (3)
C1—N1—C8—C9	−178.8 (3)	C14—C17—C22—C23	1.2 (4)
C9—O1—C10—C11	−171.6 (2)	C13—C14—C15—C16	1.7 (5)
C16—C11—C10—O1	8.7 (4)	C17—C14—C15—C16	−178.0 (3)
C12—C11—C10—O1	−174.3 (2)	C11—C16—C15—C14	−0.4 (5)
C10—O1—C9—C7	−0.4 (4)	C15—C14—C13—C12	−1.2 (5)
C10—O1—C9—C8	−179.8 (2)	C17—C14—C13—C12	178.6 (3)
N1—C8—C9—O1	3.3 (3)	C14—C13—C12—C11	−0.7 (5)
C4—C8—C9—O1	−176.8 (2)	C16—C11—C12—C13	2.0 (4)
N1—C8—C9—C7	−176.2 (3)	C10—C11—C12—C13	−175.2 (3)
C4—C8—C9—C7	3.7 (4)	C4—C5—C6—C7	0.8 (5)
N1—C8—C4—C5	177.1 (3)	C9—C7—C6—C5	0.2 (5)
C9—C8—C4—C5	−2.7 (4)	C1—C2—C3—C4	0.4 (5)
N1—C8—C4—C3	−2.5 (4)	C5—C4—C3—C2	−178.1 (3)
C9—C8—C4—C3	177.6 (2)	C8—C4—C3—C2	1.5 (4)
C12—C11—C16—C15	−1.4 (5)	C8—N1—C1—C2	0.9 (5)
C10—C11—C16—C15	175.6 (3)	C3—C2—C1—N1	−1.8 (5)
C18—C17—C14—C15	111.6 (4)	C21—C22—C23—N2	54 (24)
C22—C17—C14—C15	−71.0 (4)	C17—C22—C23—N2	−125 (23)
C18—C17—C14—C13	−68.1 (4)	C17—C22—C21—C20	−0.5 (5)
C22—C17—C14—C13	109.3 (3)	C23—C22—C21—C20	−179.0 (3)
O1—C9—C7—C6	178.1 (3)	C22—C17—C18—C19	0.7 (5)
C8—C9—C7—C6	−2.5 (4)	C14—C17—C18—C19	178.3 (3)
C3—C4—C5—C6	−179.8 (3)	C22—C21—C20—C19	0.0 (5)
C8—C4—C5—C6	0.5 (4)	C21—C20—C19—C18	0.9 (6)
C18—C17—C22—C21	0.1 (4)	C17—C18—C19—C20	−1.3 (6)
C14—C17—C22—C21	−177.4 (3)		

## References

[bb1] Chen, L. Z., Huang, Y., Xiong, R. G. & Hu, H. W. (2010). *J. Mol. Struct.* **963**, 16–21.

[bb2] Hang, T., Fu, D. W., Ye, Q. & Xiong, R. G. (2009). *Cryst. Growth Des.* **5**, 2026–2029.

[bb3] Rigaku (2005). *CrystalClear.* Rigaku Corporation, Tokyo, Japan.

[bb4] Sheldrick, G. M. (2008). *Acta Cryst.* A**64**, 112–122. 10.1107/S0108767307043930 18156677

[bb5] Xiong, R. G., Xue, X., Zhao, H., You, X. Z., Abrahams, B. F. & Xue, Z. L. (2002). *Angew. Chem. Int. Ed.* **41**, 3800–3803. 10.1002/1521-3773(20021018)41:20<3800::AID-ANIE3800>3.0.CO;2-3 12386852

[bb6] Ye, Q., Song, Y. M., Wang, G. X., Chen, K., Fu, D. W., Chan, P. W. H., Zhu, J. S., Huang, S. D. & Xiong, R. G. (2006). *J. Am. Chem. Soc.* **128**, 6554–6556. 10.1021/ja060856p 16704244

